# 

**Published:** 1886-10

**Authors:** 


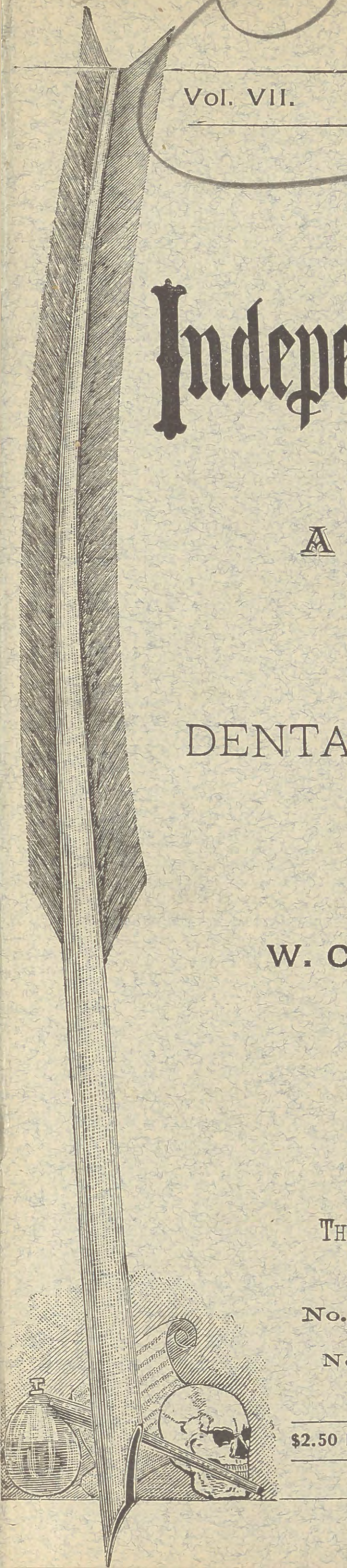

Vol. VII. OCTOBER, 1886. No. 10.

LIBRARY
INDEXED
S.G.O.

### THE Independent Practitioner:

A MONTHLY JOURNAL

DEVOTED TO

### DENTAL AND ORAL SCIENCE.

---

W. C. BARRETT, M. D., D. D. S.,

EDITOR.

---

THE NEW YORK DENTAL JOURNAL ASSOCIATION,
PUBLISHERS,
No. 35 West 46th Street, New York.
AND
No. 208 Franklin St., Buffalo, N. Y.

$2.50 Per Annum in Advance, - - - - Single Copies, 25 Cents.

Entered at the Post-Office, Buffalo, N. Y., as Second-Class matter.

